# Supplementary material for: Topological Supercavity Resonances in the Finite System
Source: Adv Sci (Weinh). 2022 May 13;9(20):2200257. doi: 10.1002/advs.202200257 (PMC9284153; doi:10.1002/advs.202200257)
Supplement: Supplementary file 1 — Supporting Information [file ADVS-9-2200257-s001.pdf]

ADVANCED SCIENCE
Open Access

Supporting Information

for *Adv. Sci.*, DOI 10.1002/advs.202200257

Topological Supercavity Resonances in the Finite System

Lujun Huang, Bin Jia, Yan Kei Chiang, Sibo Huang, Chen Shen, Fu Deng, Tianzhi Yang, David A Powell, Yong Li* and Andrey E Miroshnichenko**

Supporting Information

Topological Supercavity Resonances In The Finite System

Lujun Huang[1], Bin Jia[2], Yan Kei Chiang[1], Sibo Huang[2], Chen Shen[3], Fu Deng[1], Tianzhi Yang[4], David A Powell[1], Yong Li[2*], and Andrey E Miroshnichenko[1*]*

1 School of Engineering and Information Technology, University of New South Wales, Canberra, Northcott Drive, ACT, 2600, Australia

2 Institute of Acoustics, Tongji University, Shanghai, 200092, People's Republic of China

3 Department of Mechanical Engineering, Rowan University, Glassboro, NJ, 08028, USA

4 School of Mechanical Engineering and Automation, Northeastern University, Shenyang, 110819 China

E-mail:

lujun.huang@unsw.edu.au, yongli@tongji.edu.cn, andrey.miroshnichenko@unsw.edu.au

Section 1-Second example of coupling term for merged BICs

$$C_2(k_0d) = \gamma\{-A + B_1[1 - \cos(k_0d - k_0d_1)][1 - \cos(k_0d - k_0d_2)]\}(0 < k_0d \leq \pi) \quad (1a)$$

$$C_2(k_0d) = \gamma\{+A - B_2[1 - \cos(k_0d - k_0d_3)][1 - \cos(k_0d - k_0d_4)]\}(\pi < k_0d \ll 2\pi) \quad (1b)$$

where A=1 before BIC merging and BIC merging, and A=1-δ after BIC merging (δ is a small perturbation term). It is necessary to point out that there is an additional requirement $k_0d_3 = k_0d_1 + \pi$ and $k_0d_4 = k_0d_2 + \pi$. B_1 and B_2 can be obtained based on the continuity condition at $k_0d = \pi$, which has following forms

$$B_1 = -A/[1 - \cos(\pi - k_0d_1)][1 - \cos(\pi - k_0d_2)] \quad (2a)$$

$$B_2 = -A/[1 - \cos(\pi - k_0d_3)][1 - \cos(\pi - k_0d_4)] \quad (2b)$$

Fig.S4a plots two sets of C_2 defined by Eq. (3) in the main text and Eq. (1) in the supplementary material. It can be found that these two functions show a small deviation. Fig.4b shows the C_2 function with different k_0d_1 and k_0d_2. Fig.4c-d shows the Q-factors of antibonding and bonding modes as a function of the propagation phase k_0d.

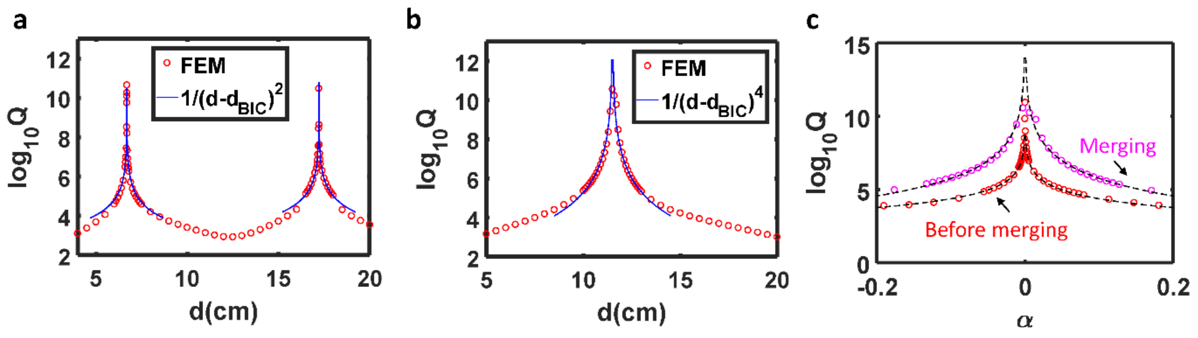

Figure S1. Q-factor Fitting for BICs. a, Q-factor Fitting for BICs before merging. b, Q-factor Fitting for Merged BIC. c, Comparison of Q factors for BIC before merging and merged BIC vs perturbation.

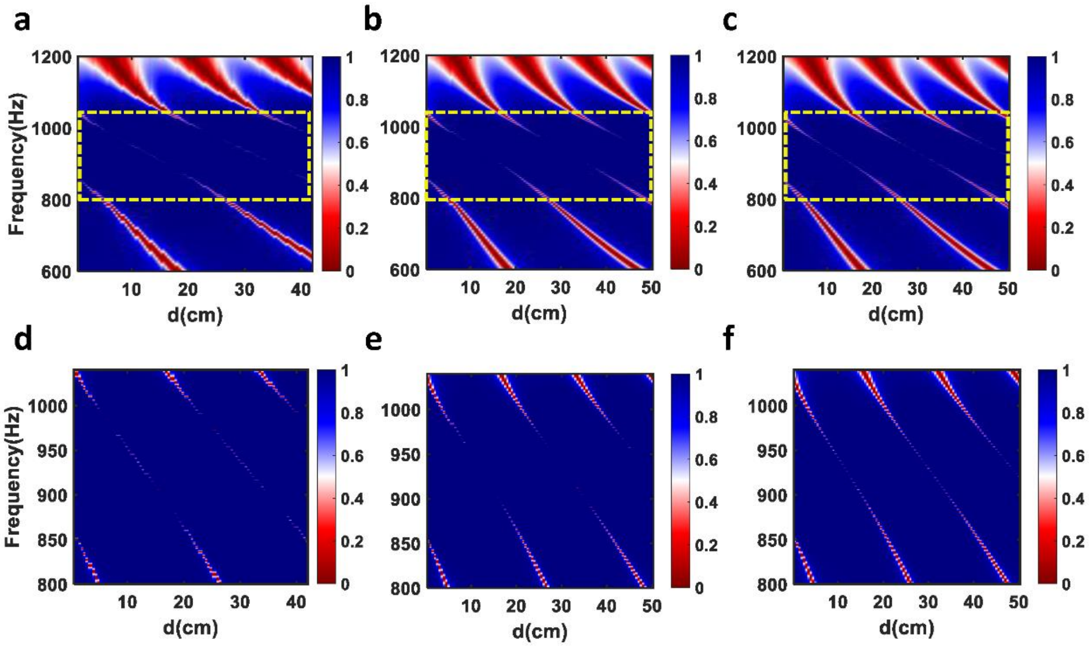

Figure S2. Calculated reflection mapping vs distance between two resonators before and after BIC merging. a-c, Reflection mapping for L=20cm (a), L=20.032cm (b), L=20.5cm (c). d-f, zoom in reflection mapping for L=20cm (d), L=20.032cm (e), L=20.5cm (f).

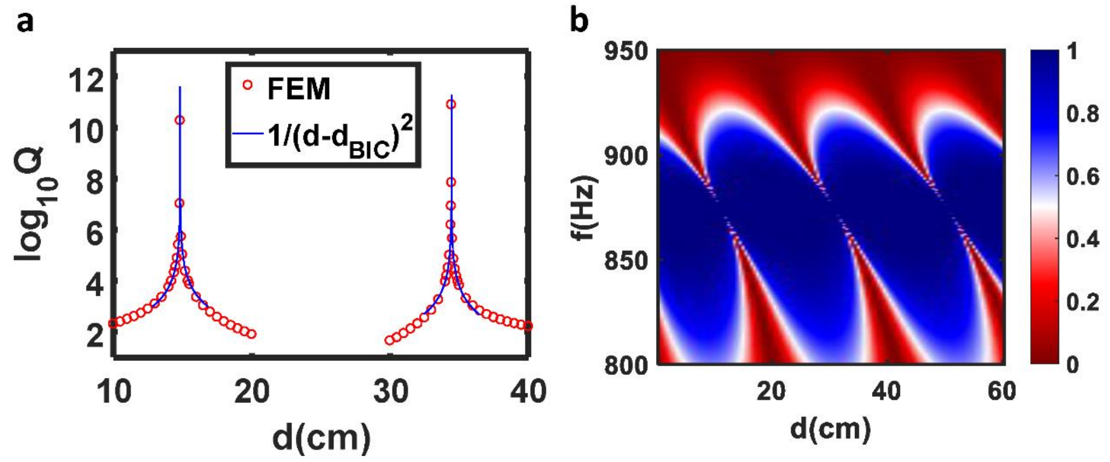

Figure S3. Fabry-Perot BIC. a, Q-factor fitting for Fabry-Perot BIC. b, Reflection mapping vs distance between two resonators

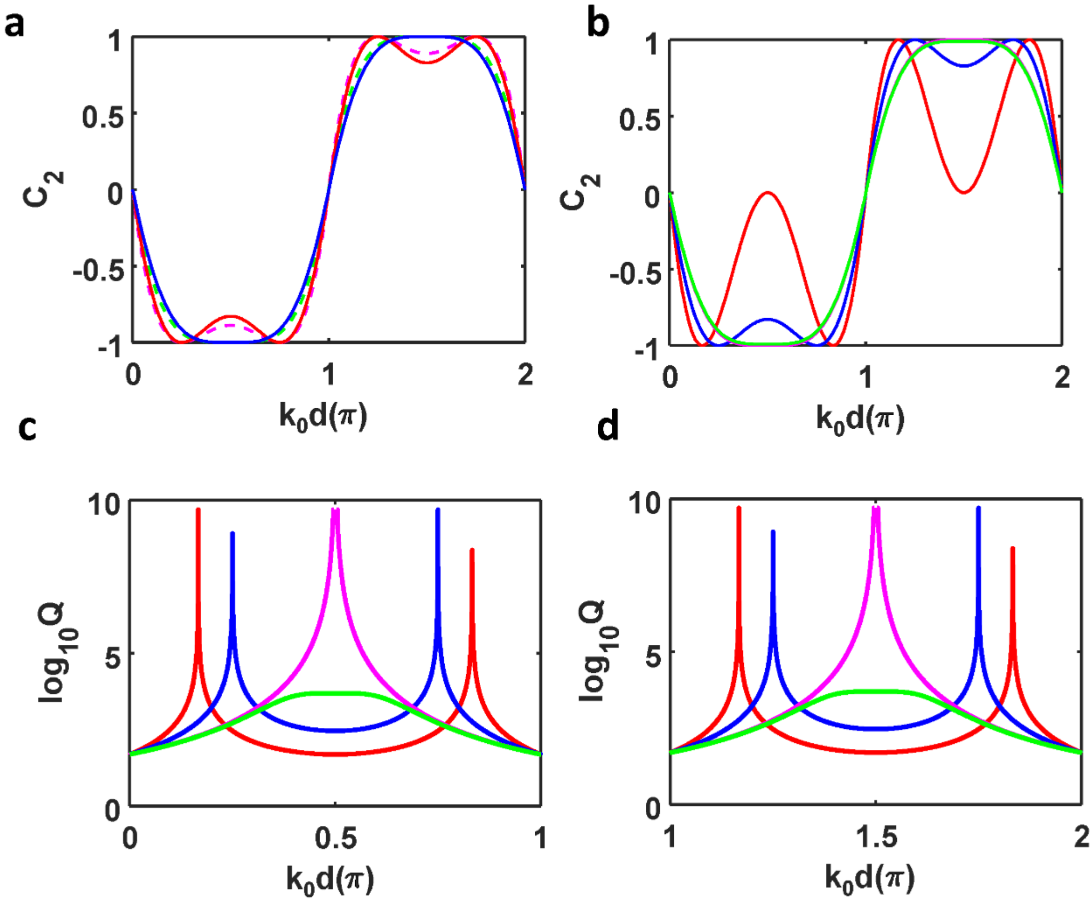

Figure S4. Coupled mode theory for modelling the merged BICs. a, C_2 as a function of the propagation phase k_0d. Dashed lines correspond to C_2 defined by Eq. (3) in the main text. Solid lines correspond to C_2 defined by Eq. (1) in supporting information. Solid red and dashed magenta lines represent $k_0d_1=\pi/4$ and $k_0d_2=3\pi/4$. Solid blue and dashed green lines represent $k_0d_1= k_0d_2=\pi/2$. b, the imaginary part of coupling C_2 vs propagation phase k_0d for merged BICs. Note that red line represents $k_0d_1=\pi/6$ and $k_0d_2=5\pi/6$, blue line represents $k_0d_1=\pi/4$ and $k_0d_2=3\pi/4$, magenta line represents $k_0d_1= k_0d_2=\pi/2$, green line represents $k_0d_1= k_0d_2=\pi/2$ and A=0.99. c-d, Q-factors of antibonding mode (c) and bonding mode (d) vs k_0d.

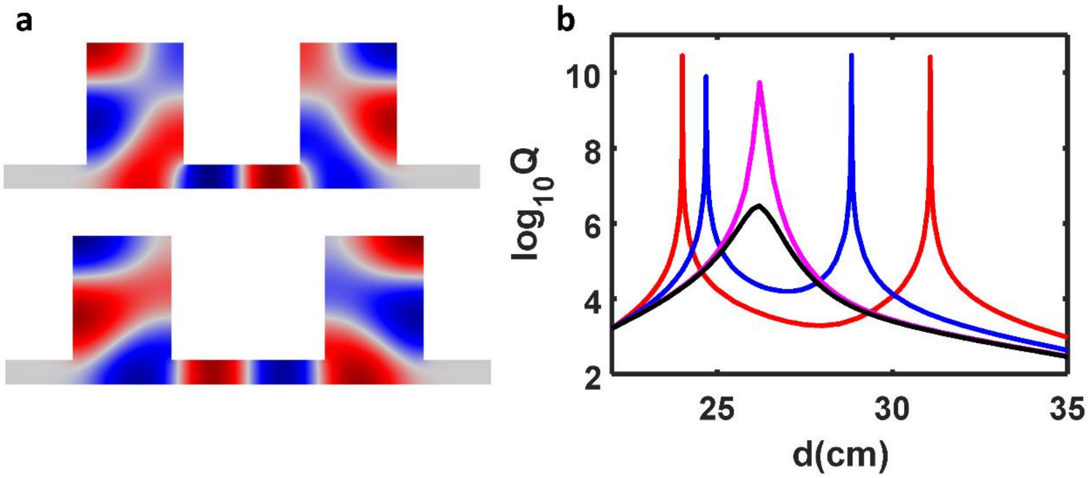

Figure S5. Antibonding merged BIC. a, Pressure field distribution for antibonding BICs. b, Q-factor for antibonding mode as a function of distance between two resonators for L=20cm (red), 20.4cm(blue), 20.674cm(magenta) and 20.7cm(black).

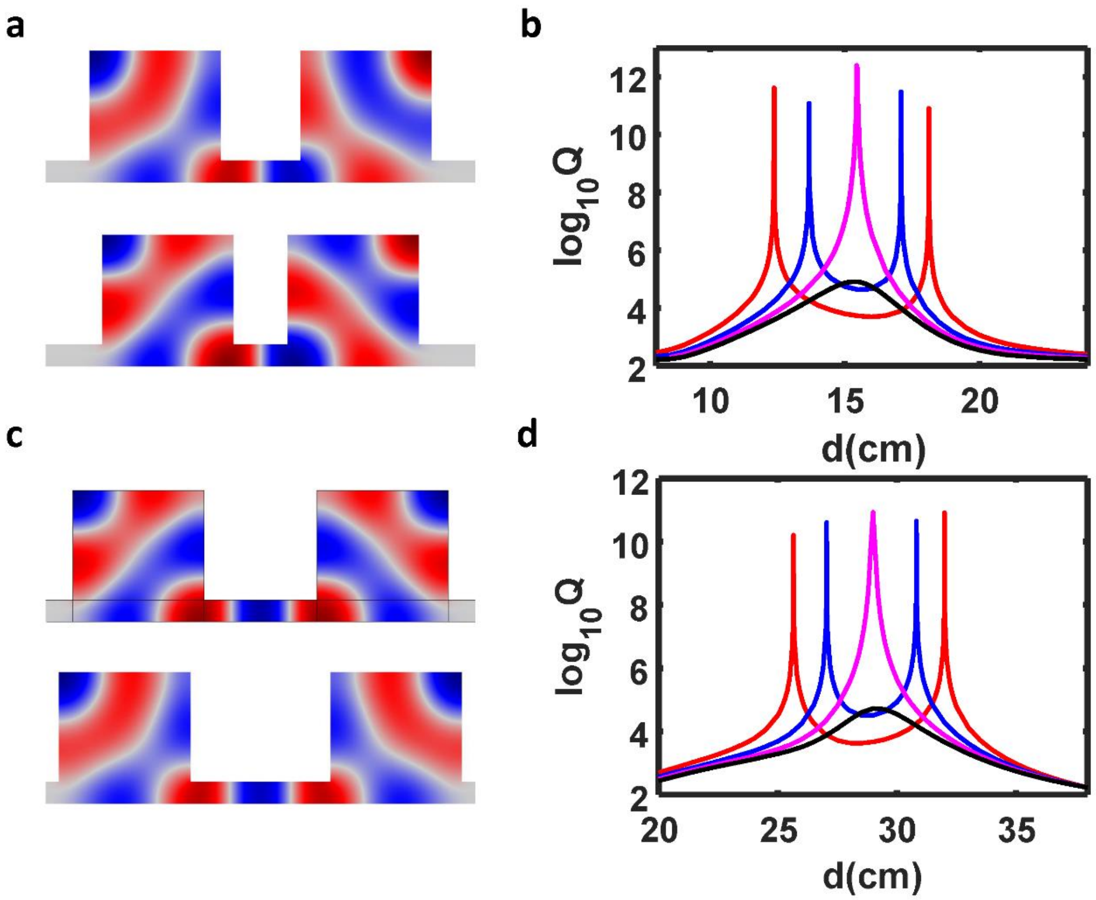

Figure S6. 3rd example of antibonding and bonding merged BICs. a, Pressure field distribution for antibonding BICs. b, Q-factor for antibonding mode as a function of distance between two resonators for width L=30cm(red), L=29.8cm(blue), L=29.6854cm(magenta), L=29.6cm(black) while the height of single resonator is set as 30cm. c, Pressure field distribution for bonding BICs. d, Q-factor for bonding mode as a function of distance between two resonators for width L=30cm(red), L=29.8cm(blue), L=29.6854cm(magenta), L=29.6cm(black).

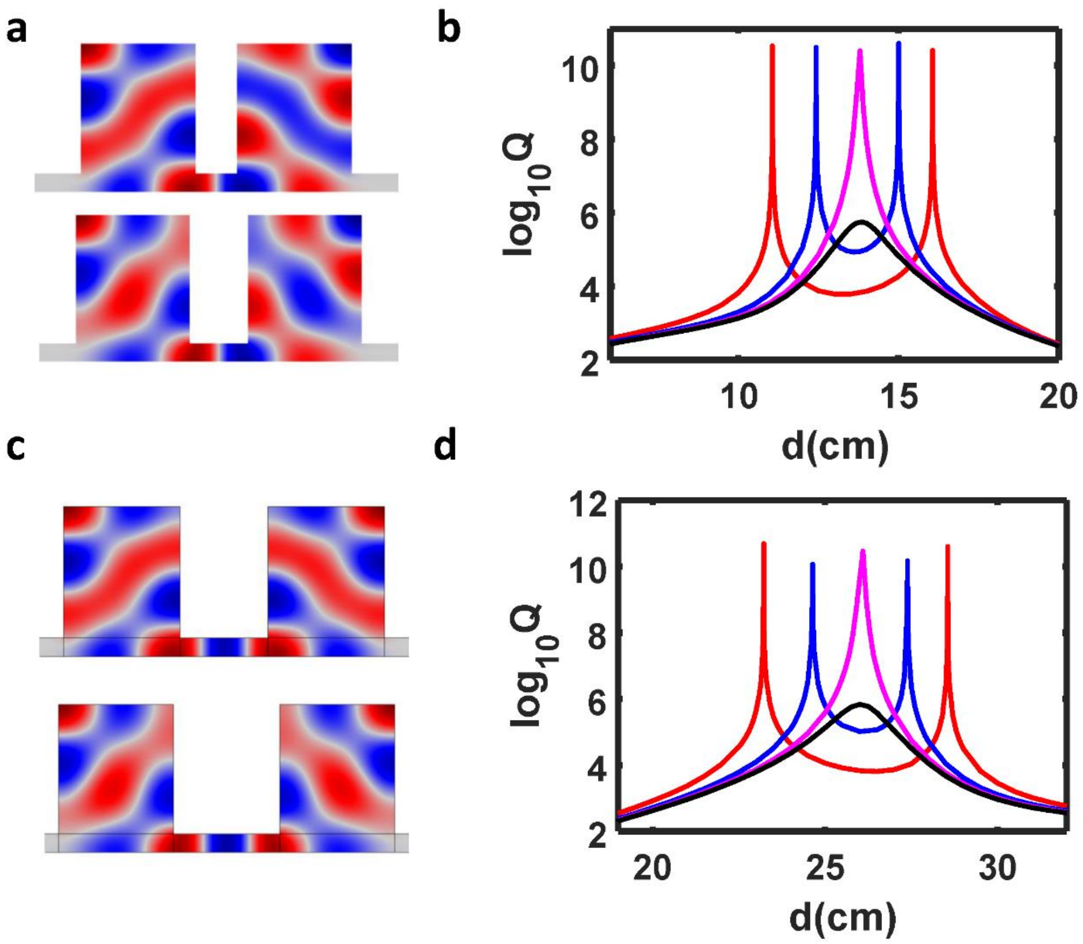

Figure S7. 4th example of antibonding and bonding merged BICs. a, Pressure field distribution for antibonding BICs. b, Q-factor for antibonding mode as a function of distance between two resonators for width L=31cm(red), L=30.8cm(blue), L=30.728cm(magenta), L=30.7cm(black) while the heigh of each resonator is set as 40cm. c, Pressure field distribution for bonding BICs. d, Q-factor for bonding mode as a function of distance between two resonators for width L=31cm(red), L=30.8cm(blue), L=30.728cm(magenta), L=30.7cm(black).

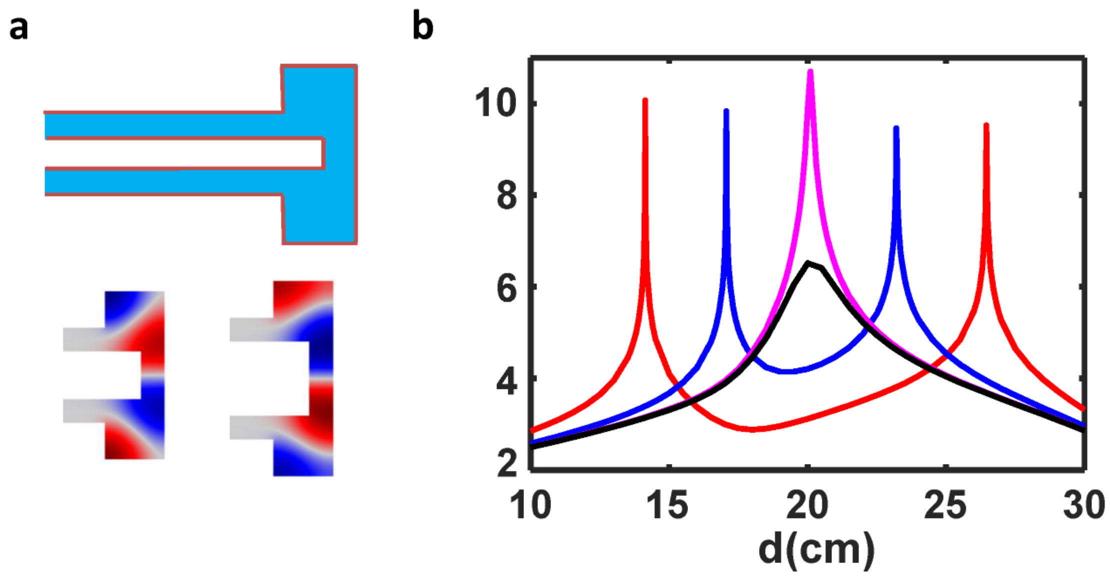

Figure S8. Equivalent structure for realizing merged BICs. a, the top panel is a schematic drawing of coupled-resonator system that are arranged side-by-side vertically. The bottom panel is the pressure field distribution of bonding BICs. b, Q-factor for bonding mode as a function of distance between two resonators for width L=20.4cm(red), L=20.1cm(blue), L=19.9968cm(magenta), L=19.99cm(black).

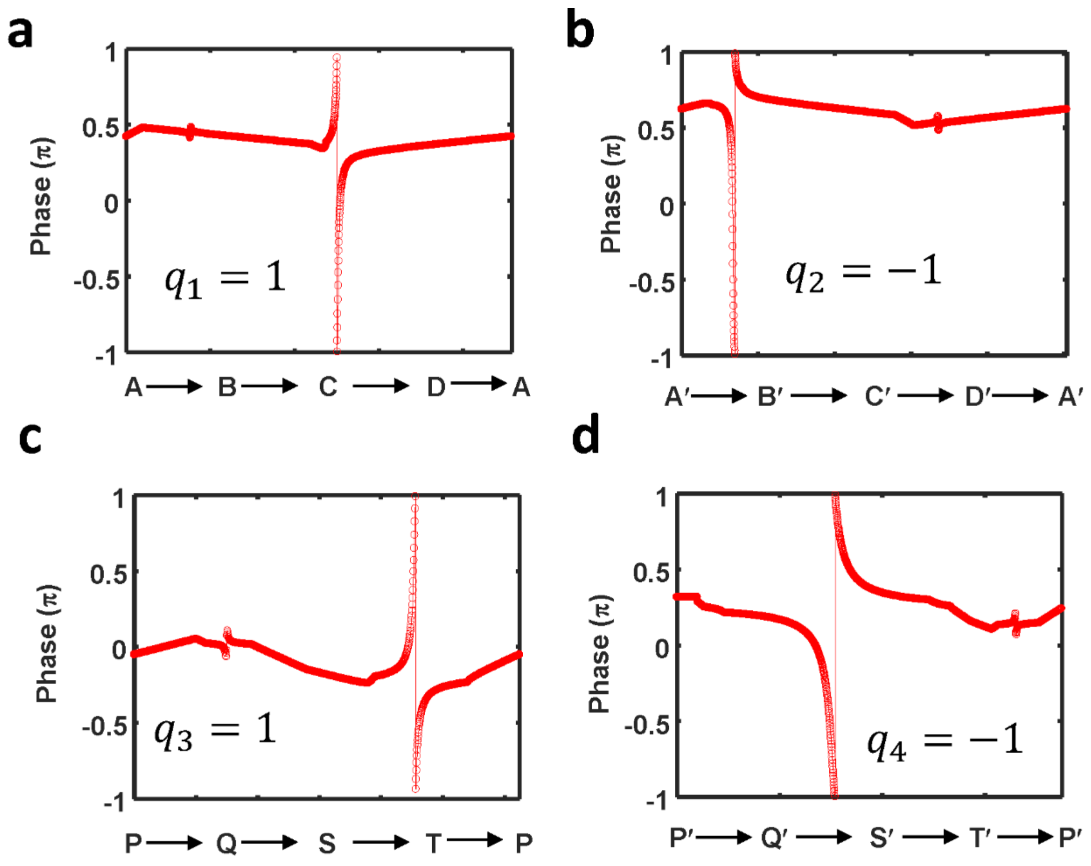

Figure S9. Topological explanation of merged BICs. a, Phase along a closed path encircling phase vortex 1. b, Phase along a closed path encircling phase vortex 2. c, Phase along a closed path encircling phase vortex 3. d, Phase along a closed path encircling phase vortex 4.

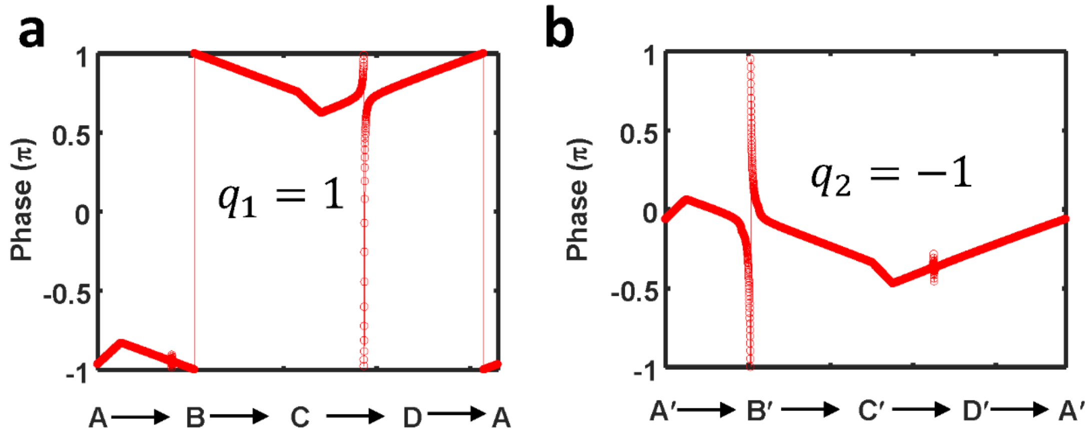

Figure S10. Topological explanation of merged BICs. a, Phase along a closed path encircling phase vortex 5. b, Phase along a closed path encircling phase vortex 6.

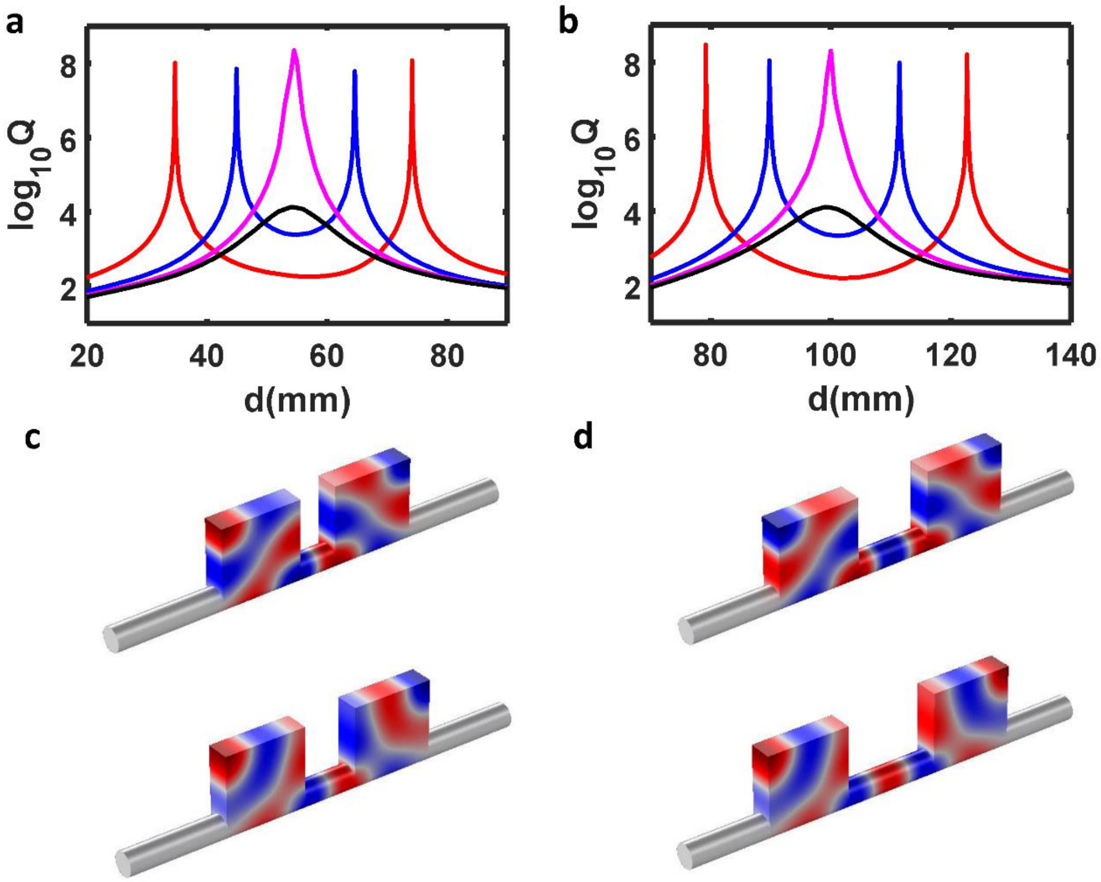

Figure S11. 2nd example of antibonding and bonding merged BICs in 3D coupled resonators-circular waveguide system. a, Q-factor for antibonding mode as a function of distance between two resonators for width L=104mm(red), L=100mm(blue), L=98.6mm(magenta). L=98mm(black). The width of the square waveguide is 30mm. b, Q-factor for bonding mode as a function of distance between two resonators for width L=104mm(red), L=100mm(blue), L=98.6mm(magenta). L=98mm(black). c, Pressure field distribution for antibonding BICs. d, Pressure field distribution for bonding BICs.

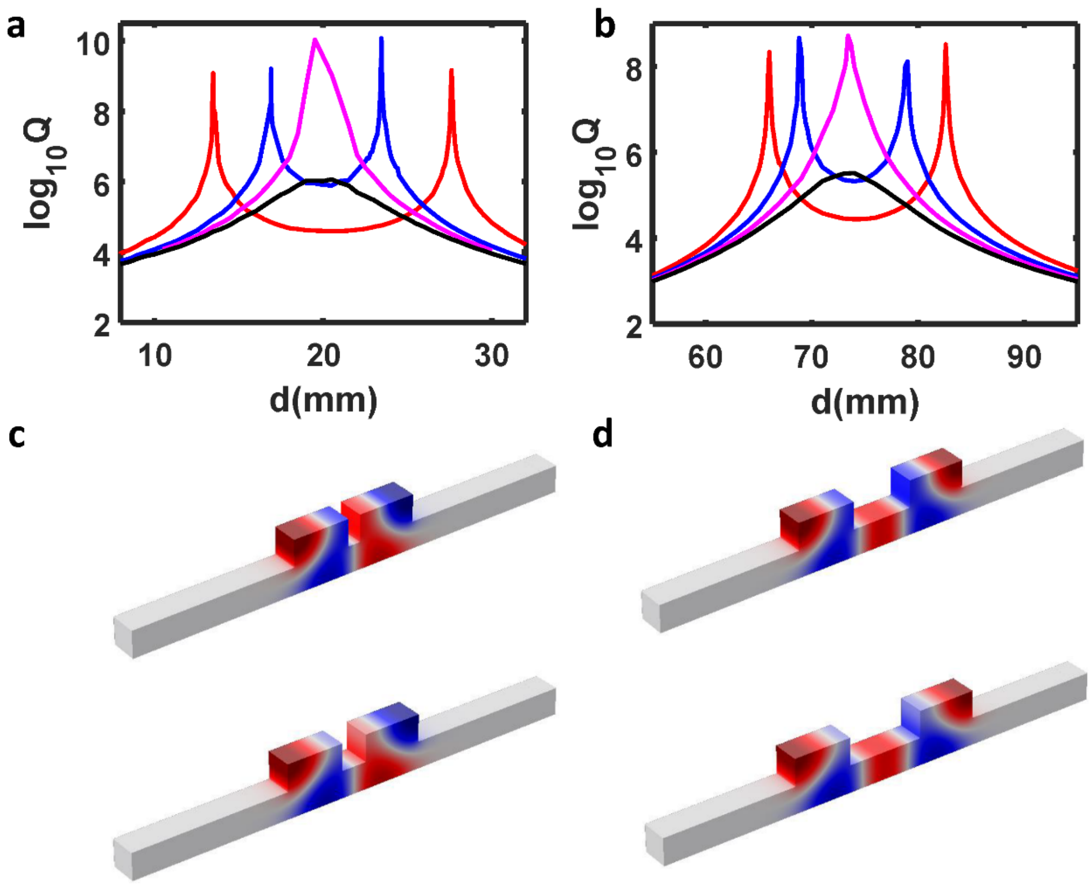

Figure S12. 1st example of antibonding and bonding merged BICs in 3D coupled resonators-rectangular waveguide system. a, Q-factor for antibonding mode as a function of distance between two resonators for width L=63mm(red), L=63.2mm(blue), L=63.254mm(magenta), L=63.3mm(black). The width of the square waveguide is 30mm. b, Q-factor for bonding mode as a function of distance between two resonators for width L=63mm(red), L=63.2mm(blue), L=63.254mm(magenta), L=63.3mm(black). c, Pressure field distribution for antibonding BICs. d, Pressure field distribution for bonding BICs.

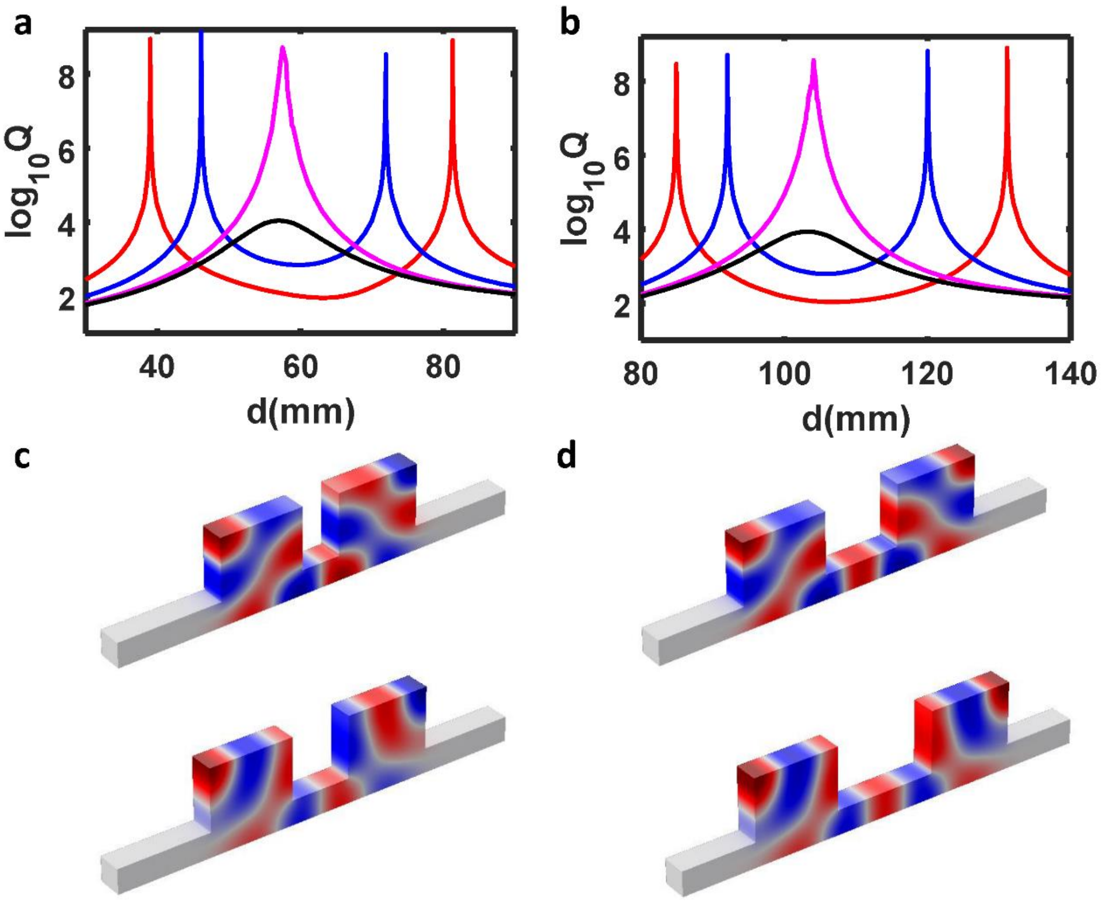

Figure S13. 2nd example of antibonding and bonding merged BICs in 3D coupled resonators-rectangular waveguide system. a, Q-factor for antibonding mode as a function of distance between two resonators for width L=106mm(red), 102mm(blue), 99.588mm(magenta), 99mm(black). The width of the square waveguide is 30mm. b, Q-factor for bonding mode as a function of distance between two resonators for width L=106mm(red), 102mm(blue), 99.588mm(magenta), 99mm(black). c, Pressure field distribution for antibonding BICs. d, Pressure field distribution for bonding BICs.

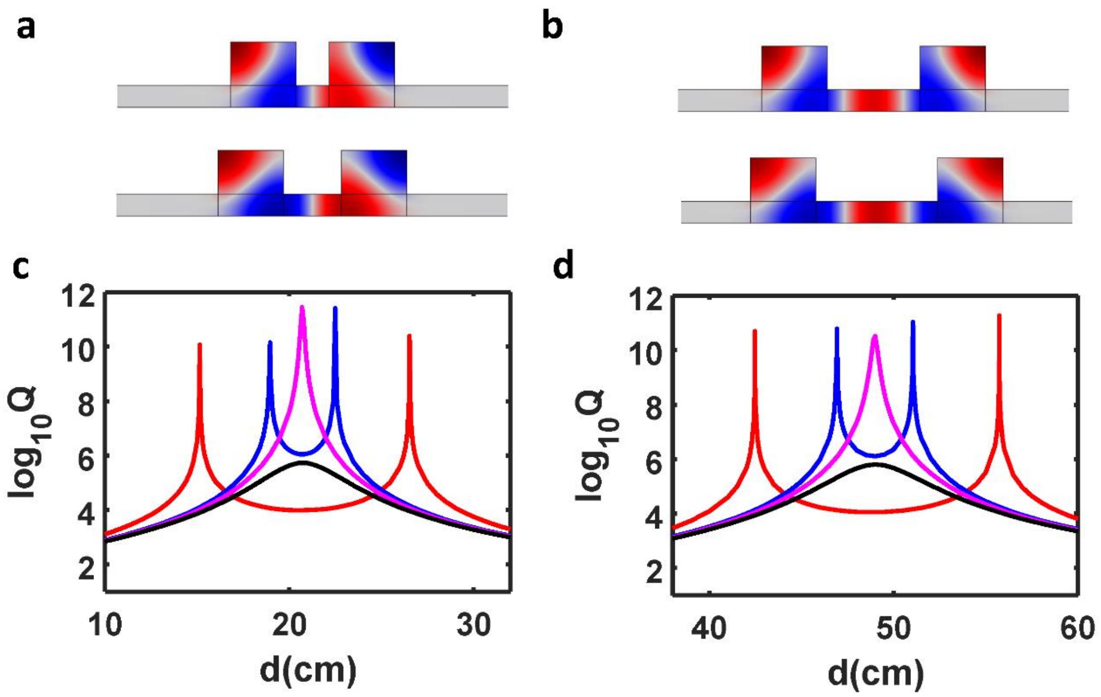

Figure S14. BICs in the photonic waveguide system for TE polarization (E//z). a-b, Ez distribution for two antibonding (a) and two bonding (b) BICs. c, Q-factor for antibonding mode as a function of distance between two photonic resonators for width L=30cm (red), L=30.16cm (blue), L=30.1765cm (magenta), L=30.2cm(black), while the heigh of single resonator and waveguide is fixed as 30cm and 10cm, respectively. d, Q-factor for bonding mode as a function of distance between two photonic resonators for width L=30cm (red), L=30.16cm (blue), L=30.1767cm (magenta), L=30.2cm(black).

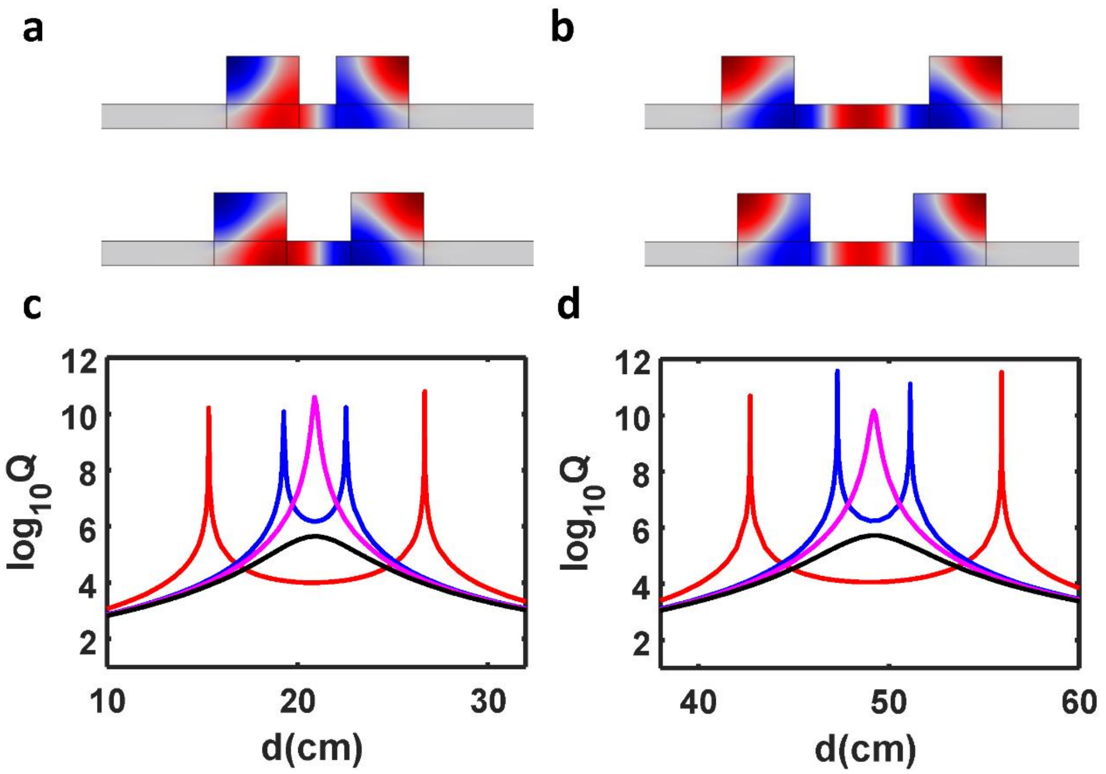

Figure S15. BICs in the photonic waveguide system for TM polarization (H//z). a-b, Hz distribution for two antibonding (a) and two bonding (b) BICs. c, Q-factor for antibonding mode as a function of distance between two photonic resonators for width L=30cm (red), L=30.16cm (blue), L=30.1742cm (magenta), L=30.2cm(black), while the heigh of single resonator and waveguide is fixed as 30cm and 10cm, respectively. d, Q-factor for bonding mode as a function of distance between two photonic resonators for width L=30cm (red), L=30.16cm (blue), L=30.1744cm (magenta), L=30.2cm(black).
